# A COVID-19 mRNA vaccine encoding SARS-CoV-2 virus-like particles induces a strong antiviral-like immune response in mice

**DOI:** 10.1038/s41422-020-00392-7

**Published:** 2020-08-17

**Authors:** Jing Lu, Guoliang Lu, Shudan Tan, Jia Xia, Hualong Xiong, Xiaofei Yu, Qingqing Qi, Xiang Yu, Li Li, Hang Yu, Ningshao Xia, Tianying Zhang, Yingjie Xu, Jinzhong Lin

**Affiliations:** 1 State Key Laboratory of Genetic Engineering, School of Life Sciences, Zhongshan Hospital, Fudan University, Shanghai, 200438 China; 2 Department of Urology, Xinhua Hospital, Shanghai Jiao Tong University School of Medicine, Shanghai, 200092 China; 3 State Key Laboratory of Molecular Vaccinology and Molecular Diagnostics, National Institute of Diagnostics and Vaccine Development in Infectious Diseases, School of Life Sciences and School of Public Health, Xiamen University, Xiamen, Fujian 361102 China; 4 State Key Laboratory of Genetic Engineering, School of Life Sciences, Fudan University, Shanghai, 200438 China; 5 Shanghai Key Laboratory for Tumor Microenvironment and Inflammation, Department of Biochemistry and Molecular Cell Biology, Shanghai Jiao Tong University School of Medicine, Shanghai, 200025 China; 6 Shanghai RNACure Biopharma Co., Ltd, Shanghai, 200438 China

**Keywords:** Immunology, Biological techniques

Dear Editor,

Since the beginning of this century, humanity has been struck three times by the coronavirus outbreak. The most recent one is caused by the SARS-CoV-2 virus, which was first reported in January 2020 and spread rapidly worldwide, developing into a global coronavirus disease pandemic coded COVID-19.^1^ By July 28, 2020, SARS-CoV-2 has caused over sixteen million COVID-19 cases worldwide and 650,805 deaths.^2^ Such a grave situation has made the development of a COVID-19 vaccine imperative and urgent.^3^

In this study, we designed three mRNA vaccine candidates for COVID-19, and they encode various forms of antigens in vaccinated hosts (Fig. 1a). RQ3011-RBD encodes the receptor-binding domain of the S (spike) glycoprotein (residues 331–524) of SARS-CoV-2 with an N-terminal signal peptide and a C-terminal membrane-anchoring helix. Vaccine RQ3012-Spike encodes the full-length wild-type S, while RQ3013-VLP is formulated from a cocktail of mRNAs encoding three structural proteins: S, M (membrane), and E (envelope) to form SARS-CoV-2 virus-like particles (VLPs). To increase the expression capacity of mRNA vaccines, all mRNAs were subjected to an in-depth sequence optimization procedure of two parameters: codons in the DNA template and modified nucleotides incorporated into mRNA. We designed ten coding sequences of the S gene (3822 nucleotides in length) with varying GC-content, maintaining the maximum codon adaptation index. For each DNA template, we tested six mRNA species with various modified nucleotides. The mRNA candidates (total of 60) displayed a considerable variation in their abilities to express S in HEK 293A cells (Supplementary information, Fig. S1a). Notably, the incorporation of pseudouridine consistently improves the expression of S, regardless of the codon sequence used. For M and E, which are relatively small proteins, we designed one codon-optimized sequence for each and screened for the optimal choice of modified nucleotides. The final mRNAs in vaccines have an optimal combination of codon and modified nucleotides that give the most robust expression (Fig. 1c, d).

**Fig. 1:**
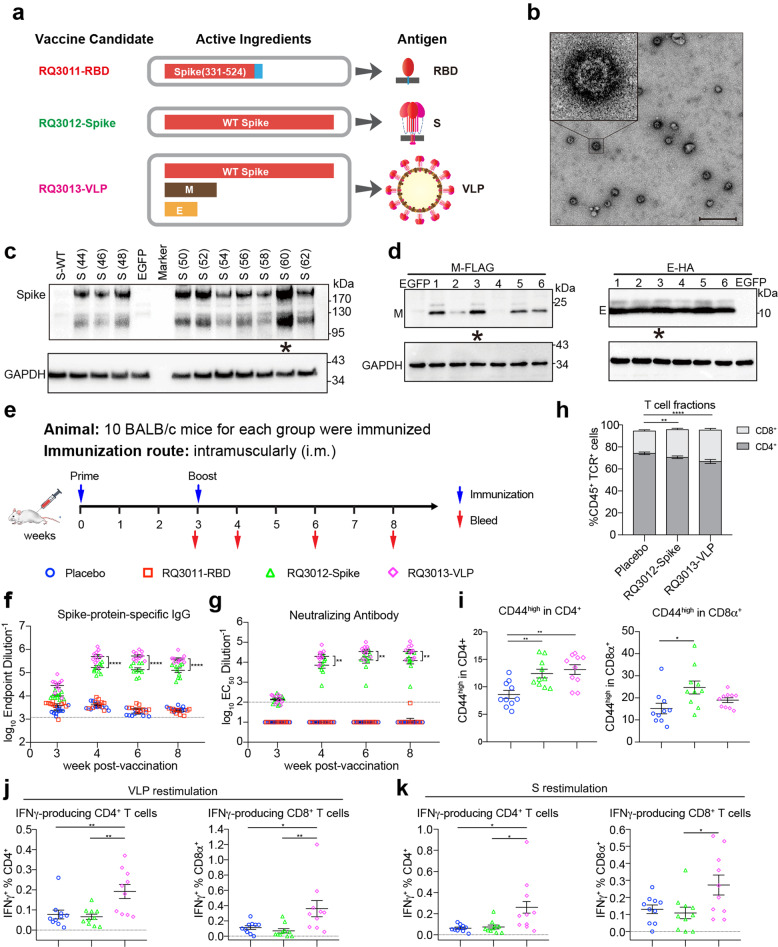
Humoral and cellular immune responses of COVID-19 mRNA LNP vaccine candidates. **a** The three mRNA vaccine candidates for COVID-19. RQ3011-RBD contains an mRNA encoding the receptor binding domain of S with a C-terminal membrane-anchoring helix. RQ3012-Spike contains an mRNA encoding the full-length wild-type (WT) S. RQ3013-VLP contains three mRNAs encoding S, M, and E proteins that can assemble into VLPs. **b** Electron microscopy images of VLPs produced by RQ3013-VLP. VLPs in the cell culture supernatant were purified and concentrated by ultracentrifugation and subjected to negative staining for electron microscopy. Scale bar, 500 nm. **c** The expression of S with various codons. GC-content for each sequence is indicated in the parenthesis. All S mRNAs have uridine replaced by Ψ. **d** Screening for optimal modified nucleotides in M and E mRNAs. A codon-optimized sequence for each gene was tested for protein expression with six different kinds of modified nucleotides. They are uridine (lane 1), m^5^C/Ψ (lane 2), Ψ (lane 3), mo^5^U (lane 4), m^1^Ψ (lane 5), and m^5^C (lane 6). All mRNAs were used to transfect HEK 293A cells, and protein expression was detected by western blotting. EGFP mRNA was used as a negative control. The mRNAs used in vaccines are indicated by stars. **e** The scheme of mice immunization. Mice (*n* = 10 per group) were either mock-immunized with Placebo (empty LNP, blue circle) or vaccinated with RQ3011-RBD (red square), RQ3012-Spike (green triangle), and RQ3013-VLP (purple diamond) intramuscularly. Time points of vaccination and bleeding are indicated by arrows. **f** The antibody response was analyzed by ELISA using the S antigen. The black dashed line indicates the titer of pre-immune mice (*n* = 10). **g** Neutralizing antibodies were analyzed by the VSV-based pseudovirus assay. Lines represent average titers of all animals in each vaccine group. The black dashed line indicates the limit of detection of the assay (reciprocal titer of 100). Any measurement below the limit of detection was assigned a value of half the limit of detection for plotting and statistical purposes. **h** Frequency changes of CD4^+^ and CD8^+^ T cells 8 weeks after vaccination. The RQ3011-RBD group was omitted for analysis. The significance of the ratio difference was indicated. **i** Frequency of CD44^high^/CD4^+^ and CD44^high^/CD8^+^ T effector cells 8 weeks after vaccination. **j**, **k** Antigen-specific responses were assessed by in vitro antigen restimulation. PBMCs isolated from the blood were stimulated with either SARS-CoV-2 VLPs or S protein and analyzed via flow cytometry for the frequency of VLP or Spike-specific CD4^+^ and CD8^+^ T cells expressing IFN-γ. Statistical analyses were carried out by Student’s *t* test when two groups were analyzed, and by ANOVA when more than two groups were analyzed (**P < 0.005; ***P < 0.001; ****P < 0.0001).

Previous studies on SARS and MERS have shown that coronavirus VLP assembly requires at least three structural proteins: S, M, and E.^4,5^ Based on our established system,^6^ we co-transfected three mRNAs encoding SARS-CoV-2 S, M, and E at a molar ratio of 1:2:2 into cells. All three proteins can be detected by western blotting in culture media.^6^ We then purified VLPs through a sucrose gradient and examined the particles under an electron microscope.^6^ The VLPs have an average diameter of 100 nm, with the spike protein densely decorating the surface, suggesting that SARS-CoV-2 virus-like particles have formed.^6^

We used the well-established lipid nanoparticles (LNPs) to package mRNAs. The mRNA encapsulation efficiency of all three LNP vaccine candidates was greater than 98%, with an average size of 100 nm in diameter (Supplementary information, Fig. S1b, c). All LNPs were able to transfect HEK 293A cells and express antigens of interest, as judged by western blotting (Supplementary information, Fig. S1d). Virus-like particles secreted into culture media of cells transfected with RQ3013-VLP can be detected by western blotting and electron microscopy (Fig. 1b).

We then assessed the immunogenicity of each mRNA LNP vaccine candidate in BALB/c mice. A group of mice (*n* = 10) were immunized intramuscularly with each of the vaccines on day 0 (Fig. 1e). Each dose of vaccine contains 2 μg of RBD mRNA for RQ3011-RBD or 6 μg of S mRNA for RQ3012-Spike. For RQ3013-VLP, each treatment delivers 6 μg of S, 2.5 μg of M, and 1.5 μg of E mRNAs. A fourth control group of mice (*n* = 10) were included in the study, for which 22 μg empty LNP was used as placebo. All groups were boosted on day 21, 3 weeks after the prime injection. No inflammation or other adverse effects were observed at the sites of injection. Sera were collected on days 20 (week 3), 28 (week 4), 42 (week 6), and 56 (week 8).

All sera were evaluated for binding to the S ectodomain by enzyme-linked immunosorbent assay (ELISA). Binding antibodies can be detected in mice immunized with RQ3012-Spike and RQ3013-VLP on days 20 after the first injection, while RQ3011-RBD showed marginal stimulation (Fig. 1f). Following a boost, the antibody titers increased dramatically in mice receiving RQ3012-Spike or RQ3013-VLP, and peaked at week 3, remaining stable at week 8. A boost did not increase the titer for RQ3011-RBD, which dropped to the level of the placebo group. Notably, mice receiving RQ3013-VLP had the strongest immune response and developed significantly higher titers of S-specific binding antibody than mice receiving RQ3012-Spike.

Since we included M and E mRNA in RQ3013-VLP, we analyzed whether M and E induced protein-specific immunoglobulin G (IgG). For that purpose, sub-VLPs consisting of M and E proteins were purified^7^ and used for ELISA. No M- or E-specific antibodies were detected in mice vaccinated with RQ3013-VLP (Supplementary information, Fig. S2c).

The presence of neutralizing antibodies (NAbs) was evaluated for all groups using our recently established pseudovirus neutralization assay for SARS-CoV-2. We and others have previously demonstrated that NAb titers measured from the vesicular stomatitis virus (VSV) pseudovirus assay correlated well with NAb titers measured from a live SARS-CoV-2 virus assay.^8,9^ In mice receiving RQ3012-Spike, the mean NAb titers (EC50) reached 10,000 at week 4, 1 week after a boost, and peaked at week 6, maintaining relatively stable at week 8 (Fig. 1g and Supplementary information, Fig. S2a, b). In mice receiving RQ3013-VLP, the mean NAb titer rose to 25,028 at week 4, 2.5-fold higher than that in the RQ3012-Spike group. By week 8, the NAb titer was still increasing, with the highest EC50 value of more than 100,000. The differences in NAb titers between RQ3012-Spike and RQ3013-VLP are significant throughout the tested weeks (*P* = 0.0021 at week 4, *P* = 0.0042 at week 6, *P* = 0.0015 at week 8). With the exception of one animal, there are no detectable NAbs in mice receiving RQ3011-RBD.

T cell response is critical for vaccine-induced cellular and humoral protection against future infection. Mice immunized with RQ3013-VLP showed altered CD4^+^:CD8^+^ T cell ratio (0.67:0.29) in the blood compared to the placebo group (0.74:0.20), with a significant increase in CD8^+^ T cell frequency, which is important for antiviral immunity (Fig. 1h). Both RQ3012-Spike and RQ3013-VLP induced comparable CD4^+^ T cell activation, while RQ3012-Spike induced slightly higher CD8^+^ T cell activation, as demonstrated by CD44 staining (Fig. 1i). However, higher CD8^+^ T cell activation in RQ3012-Spike immunized mice may not be necessarily specific to the antigen used in vaccination, as vaccines can induce numerous immune factors, such as cytokines, to influence by-stander activations of CD8^+^ T cells. To assess the vaccine-specific T cell activation, peripheral blood mononuclear cells (PBMCs) from immunized mice (RQ3012-Spike and RQ3013-VLP) at week 8 were restimulated with purified VLPs or recombinant S protein in vitro, and the IFNγ production by both CD4^+^ and CD8^+^ T cells was examined. Little VLP- and S-specific responses can be detected in T cells from mice receiving RQ3012-Spike. In contrast, vaccination by RQ3013-VLP induced robust VLP- and S-specific T cell responses in mice (Fig. 1j, k).

In this pilot study, we have tested the immunogenicity of three optimized mRNA LNP vaccine candidates for COVID-19. RQ3012-Spike and RQ3013-VLP contain the same amount of S mRNA, but only RQ3013-VLP elicited both humoral and T cell immune responses. It also developed the highest titers of NAbs, suggesting that when S is presented in secreted vesicles as VLPs, it induces a more robust immune response than when it is displayed on the cell membrane. The antiviral-like immunity elicited by RQ3013-VLP is in agreement with the previous findings that VLPs can mimic antigenic properties of authentic native viruses.^10^ Surprisingly, our RQ3011-RBD (2 μg RNA/dose) failed to induce sufficient immunity in mice and proved to be a weak candidate. However, this does not exclude the use of RBD as an antigen choice for future mRNA vaccines or other vaccine platforms. Several potential improvements might enhance the immunogenicity of an RBD-encoding mRNA vaccine. Our RQ3011-RBD presents monomeric RBD immunogen on the cell surface through an appended C-terminal transmembrane helix, which might have an effect on the conformation of RBD and limit exposure of key antigen sites on RBD to the immune system. A secreted form of RBD could be used instead that might augment the antigenicity. Recently, a DNA vaccine encoding a trimeric form of RBD confers protection against SARS-CoV-2 in rhesus macaques,^11^ and a similar design could be implemented by the mRNA platform. In summary, our data provide support for the VLP strategy when designing an mRNA vaccine for COVID-19. Echoing with the encouraging results from the phase I data of mRNA-1273, an mRNA vaccine candidate from Moderna that encodes the spike protein stabilized in the prefusion form, our results demonstrate that the mRNA platform holds great promise for a vaccine solution for COVID-19. Most importantly, the mRNA platform gives us unprecedented flexibility in vaccine design and screening for more effective candidates.

## Supplementary information

Supplementary Information
