# Supplementary material for: A COVID-19 mRNA vaccine encoding SARS-CoV-2 virus-like particles induces a strong antiviral-like immune response in mice
Source: Cell Res. 2020 Aug 17;30(10):936–9. doi: 10.1038/s41422-020-00392-7 (PMC7429369; doi:10.1038/s41422-020-00392-7)
Supplement: Supplementary file 1 — Supplementary Information [file 41422_2020_392_MOESM1_ESM.pdf]

Supplementary information

Materials and Methods

Cell lines and antibodies

HEK 293A cells were maintained in high glucose DMEM(GIBCO) supplemented with 10% FBS(GIBCO), 1% penicillin/streptomycin (P/S) (GIBCO) in a 5% CO_2 environment at 37°C. The following primary antibodies were used in this study: anti-HA-Tag (#3724, Cell Signaling Technology), anti-SARS-CoV-2 (2019-nCoV) Spike Antibody (40589-T62, Sino Biological), Anti-Flag M2 antibody (F1804, Sigma-Aldrich), and anti-GAPDH Antibody (60004, Proteintech). The secondary antibodies used were Peroxidase AffiniPure Goat Anti-Rabbit IgG (H+L) (111-035-003, Jackson ImmunoResearch), and Peroxidase AffiniPure Goat Anti-Mouse IgG (H+L) (115-035-003, Jackson ImmunoResearch).

Preparation of modified mRNAs and LNP

All mRNAs were synthesized in vitro using T7 polymerase-mediated RNA transcription. Codon-optimized open reading frames plus flanking 5' and 3' UTRs for all genes were synthesized by GenScript and cloned into a vector downstream of a T7 promoter. The DNA templates were amplified by PCR reaction, purified, and used for *in vitro* transcription (IVT). The Cap1 analog (B8176, APExBIO) was included in the IVT reaction to place a cap 1 structure at the 5' end of mRNAs. All mRNAs were synthesized using either all unmodified nucleotides or global substitutions of cytidine (C) for 5-methyl-cytidine (m^5C), or global substitutions of uridine (U) for the modified uridine analogs pseudouridine (Ψ), N1-methyl-pseudouridine ($m^1\Psi$), 5-methyoxy-uridine (mo^5U), or a combination of Ψ and 5-methyl-cytidine (m^5C). Following transcription, a poly(A)-tail was added to RNA transcripts using a poly(A) tailing kit (K1053, APExBIO). mRNA was purified by either the MegaClear kit (ThermoFisher) or the RNAClean and Concentration kit (APExBIO).

LNP formulations were prepared as described previously with a slightly modified procedure. Briefly, lipids were dissolved in ethanol at molar ratios of 50:10:38.5:1.5 (DLin-MC3-DMA: DSPC: cholesterol: PEG2000-DMG). The lipid mixture was mixed with an equal volume of mRNA solution in the 50mM citrate buffer (pH 3.0) through a T-mixer. Formulations were immediately diluted 2-fold with the 50mM citrate buffer (pH 3.0) and dialyzed against PBS (pH 7.4) in dialysis cassettes for at least 15 hours. Formulations were concentrated using Amicon Ultra Centrifugal Filters (Millipore), passed through a 0.22-μm filter. RNA encapsulation for all formulations was determined by the Quant-iT RiboGreen RNA Assay (ThermoFisher). Endotoxin were determined to be less than 1 EU/ml. Particle sizes were

analyzed in a nanoparticle tracking analysis instrument (ZetaView, Particle Metrix) and visually inspected under a 120kV cryo-electron microscopy (TALOS, ThermoFisher).

Protein expression of mRNAs and LNPs

The protein expression of mRNAs was tested in HEK 293A cells. mRNA transfection was carried out using lipofectamine 2000 (lipo2K) at a ratio of 1:2 (mRNA: lipo2K). For expression of virus-like particles (VLPs), S, M, and E mRNAs were co-transfected into HEK 293A cells at various molar ratios, and the supernatant was collected 48-hour post-transfection for analysis. For LNP，20μg of LNPs were incubated with HEK 293A cell in one well of a six-well plate for 48 hours, and cells and media were collected for protein analysis. Protein expression was detected by western blotting. Briefly, cells were collected and rinsed with PBS and lysed by lysis buffer (20mM Tris-HCl(pH=7.4), 150mM NaCl, 3mM $MgCl_2$, 1% Triton X-100) supplemented with protease inhibitor. For VLP detection, the culture media was subjected to ultracentrifugation through a 20% sucrose cushion. The pellet was resuspended in PBS. All samples were mixed with SDS loading buffer and separated in a 4-20% gradient SDS-Gel, and transferred to PVDF membranes (ThermoFisher) by Trans-Blot Turbo Transfer System (BioRad). The blots were blocked with 5% non-fat dry milk in TBST and then incubated with appropriate primary antibodies. Signals were detected with HRP-conjugated secondary antibodies and an enhanced chemiluminescence (ECL) detection system.

Negative-stain electron microscopy of VLPs

For VLP purification, cell culture media after 36-hour post-transfection were concentrated using 100-kDa cutoff concentrator (Amicon Ultra-15, Millipore) before being layered on the top of the 30-40-50% (w/v) sucrose gradient in 20 mM HEPES-Na (pH 7.4), 120 mM NaCl. The sucrose solution between 30-40% (w/v) was extracted immediately with a 5 mL syringe after ultracentrifugation for 90 minutes at 4°C, 31,000 rpm (rotor SW32, Beckman). The VLPs-containing solution was buffer-exchanged against PBS three times. To prepare grids for negative-stain transmission electron microscopy (TEM), 5 μL purified VLPs were absorbed for 2 minutes on a glow-discharged carbon-coated grid. The grid was stained in a dropwise manner for 60 seconds and loaded on the Talos L120C microscope (ThermoFisher) for visualization of VLPs.

Mice immunization

All animal experiments were performed under the ethical guidelines of Fudan University. Six-week-old female BALB/c mice (n=10 for each group) were immunized intramuscularly with 100 μL of LNP vaccine candidates or placebo at week 0. At week 3, all mice received a boost vaccination. Sera were collected from all the mice on days 0, 20, 28, 42, and 56 for analysis of binding and NAb antibody responses.

ELISA for SARS-CoV-2 S-specific IgG

SARS-CoV-2 S-specific antibody responses in immunized sera were determined by enzyme-linked immunosorbent assay (ELISA) assay, as previously described. Briefly, 96-well plates were coated with 100 μL of coating buffer containing 100ng/well Spike S1+S2 ECD-His recombinant protein (Sino Biological) at 4°C overnight. Plates were blocked with 2% bovine serum albumin solution in PBST at room temperature for 1 hour. Immunized mice sera were diluted 100-fold as the initial concentration, and then a 5-fold serial dilution of a total of 11 gradients in PBS buffer. PBST washed plates were incubated with serially diluted sera at room temperature for 2 hours. For determination of S-specific antibody response, plates were incubated with goat anti-mouse IgG HRP (Proteintech Cat: SA00001-1) at 37°C for 1 hour and then substrate tetramethylbenzidine (TMB) solution (Invitrogen) was used to develop. The color reaction was quenched with 1N sulfuric acid at about 10 minutes, and the optical density was measured at wavelength 450 nm by Synergy H1 microplate reader (BioTek).

ELISA for SARS-CoV-2 M and E-specific IgG

To prepare antigens for ELISA, we cloned SARS-CoV-2 M and E genes fused by a T2A sequence into the pCDNA3.1 vector. HEK293F cells were used to express proteins. Secreted particles formed by M and E proteins were collected from cell culture by pelleting via ultracentrifugation through a 20% sucrose cushion and further purified through a 30-50% sucrose gradient. Purified M/E particles (6 μg/well) were added to 96-well plates and incubated at 4°C overnight. The ELISA was developed and measured as described above.

Pseudovirus-based neutralization assay

The pseudovirus-based neutralization assay was performed as described[1]. BHK21-hACE2 cells were seeded in 96-well plates. Mice sera were mixed with diluted VSV-SARS-CoV-2-Sdel18 virus (MOI=0.05) and incubated at 37°C for 1h. All samples and viruses were diluted with DMEM (10%FBS), the mixture was added to seeded BHK21-hACE2 cell. After 12h incubation, fluorescence images were obtained by Opera Phenix or Operetta CLS equipment (PerkinElmer). For quantitative determination, fluorescence images were analyzed by Columbus system (PerkinElmer), and the numbers of GFP-activated cells for each well were counted to represent infection performance. The reduction (%) of mice sera treatment GFP-activated cell numbers in comparison with non-treated control well was calculated to show the neutralizing potency.

T cell restimulation assay

Blood was collected through retro-orbital bleeding with heparinized capillary tubes (Kimble Chase). Blood cells were pelleted by centrifuging at 4,000 rpm for 20 minutes at 4°C and resuspended in 1 mL RBC lysis buffer (ThermoFisher). After incubation at room temperature for 5 minutes, the cell suspension was transferred into 10 ml FACS (DPBS supplemented with

0.2% BSA, 2mM EDTA) and spun down at 400 g for 10 minutes at 4 °C. Fresh PBMCs were suspended in FACS, blocked with anti-CD16/32 antibody (BD Biosciences) at 4 °C for 30 minutes and stained with ZombieNIR Fixable Viability Dye (Biolegend) as well as antibodies against mouse CD45 (Biolegend), TCRβ (Biolegend), CD4 (ThermoFisher), CD8α (Biolegend) and CD44 (Biolegend) at 4 °C for 30 minutes. Cells were then washed twice with FACS before analyzed on a Gallios flow cytometer (Beckman).

To assess antigen-specific T cell responses, PBMCs were resuspended in complete RPMI media (RPMI 1640, 10% FBS, 1x Sodium pyruvate, 1x L-Glutamine, 1x Non-essential amino acids, 1x Penicillin-Streptomycin, 1x β-Mercaptoethanol) supplemented with 10 U/mL recombinant mouse IL-2 (Biolegend) and were stimulated with 30 μg/mL VLP and 10 μg/mL recombinant S, respectively. Cells were then cultured in a 37 °C cell incubator with 5% CO_2 for 24 hours and 5μg/mL Brefeldin A (Sigma-Aldrich) was added 6 hours before harvest. Stimulated cells were washed in ice-cold FACS and fixed in Fixation Buffer (Biolegend) for 30 minutes at 4 °C, followed by permeabilization with Intracellular Staining Permeabilization Wash Buffer (Biolegend) for 90 minutes at 4 °C. Cells were first blocked with anti-CD16/32 antibody (BD Biosciences) and stained with antibodies against mouse CD45 (Biolegend), TCRβ (Biolegend), CD4 (ThermoFisher), CD8α (Biolegend), CD3ε (ThermoFisher) and IFN-γ (Biolegend). After washing twice, cells were analyzed on a Gallios flow cytometer (Beckman).

Statistical Analysis

All data was graphed, and statistics performed using GraphPad Prsm8.0. The EC50 values were calculated by non-linear regression. Statistical analyses were carried out by Student's *t*-test when two groups were analyzed, and by ANOVA when more than two groups were analyzed.

References

1 Xiong, H.-L. *et al.* Robust neutralization assay based on SARS-CoV-2 S-bearing vesicular stomatitis virus (VSV) pseudovirus and ACE2-overexpressed BHK21 cells. *bioRxiv*, 2020.2004.2008.026948, doi:10.1101/2020.04.08.026948 (2020).

Supplementary information, Fig. S1

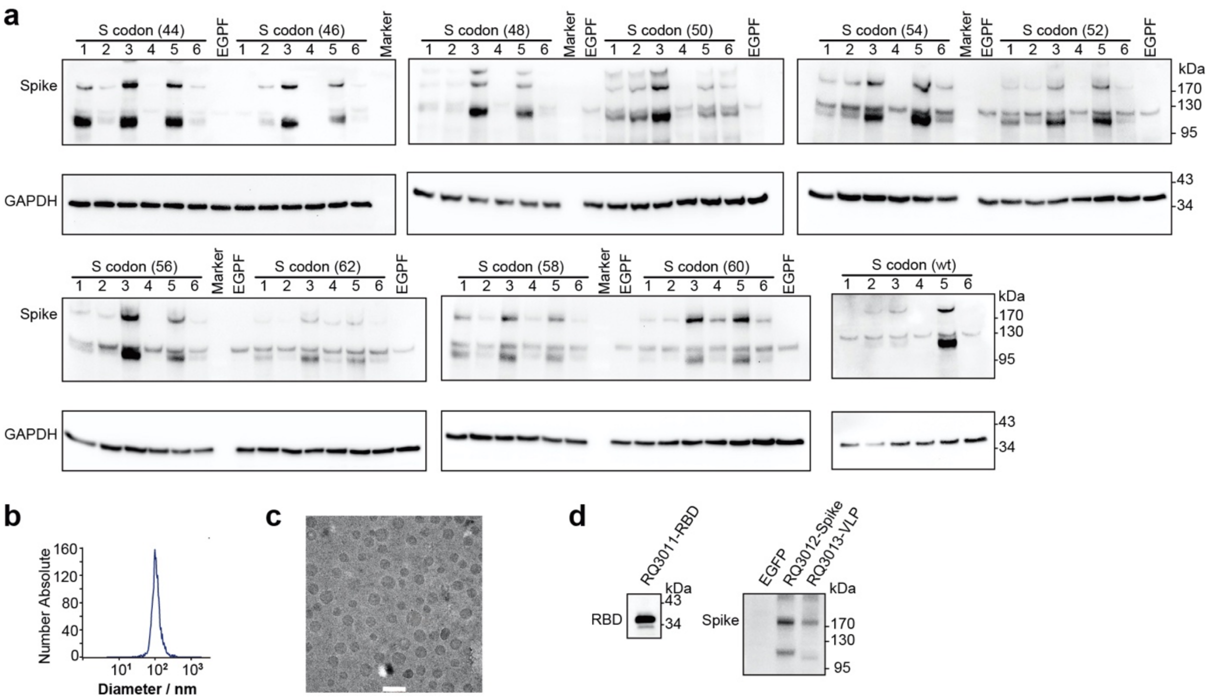

Fig. S1: mRNA sequence optimization for SARS-CoV-2 S and size distribution of LNP. **a** Screening for optimal codons and modified nucleotides for S mRNAs. Ten codon-redesigned sequences, as well as the wild-type one, were synthesized and used as templates for S mRNA transcription. Each sequence was transcribed into six mRNA species incorporating different kinds of modified nucleotides. They are uridine (lane 1), m^5C/Ψ (lane 2), Ψ (lane 3), mo^5U (lane 4), $m^1\Psi$ (lane 5), and m^5C (lane 6). mRNAs were used to transfect HEK 293A cells, and protein expression was detected by western blot. EGFP mRNA was used as a negative control. **b** A representative size distribution of the lipid nanoparticles of mRNA vaccines. **c** A cryo-EM image of the LNP vaccine candidate RQ3013-VLP (scale bar: 100 nm). **d** Expression of RBD and Spike in HEK 293A cells transfected with mRNA LNPs

Supplementary information, Fig. S2

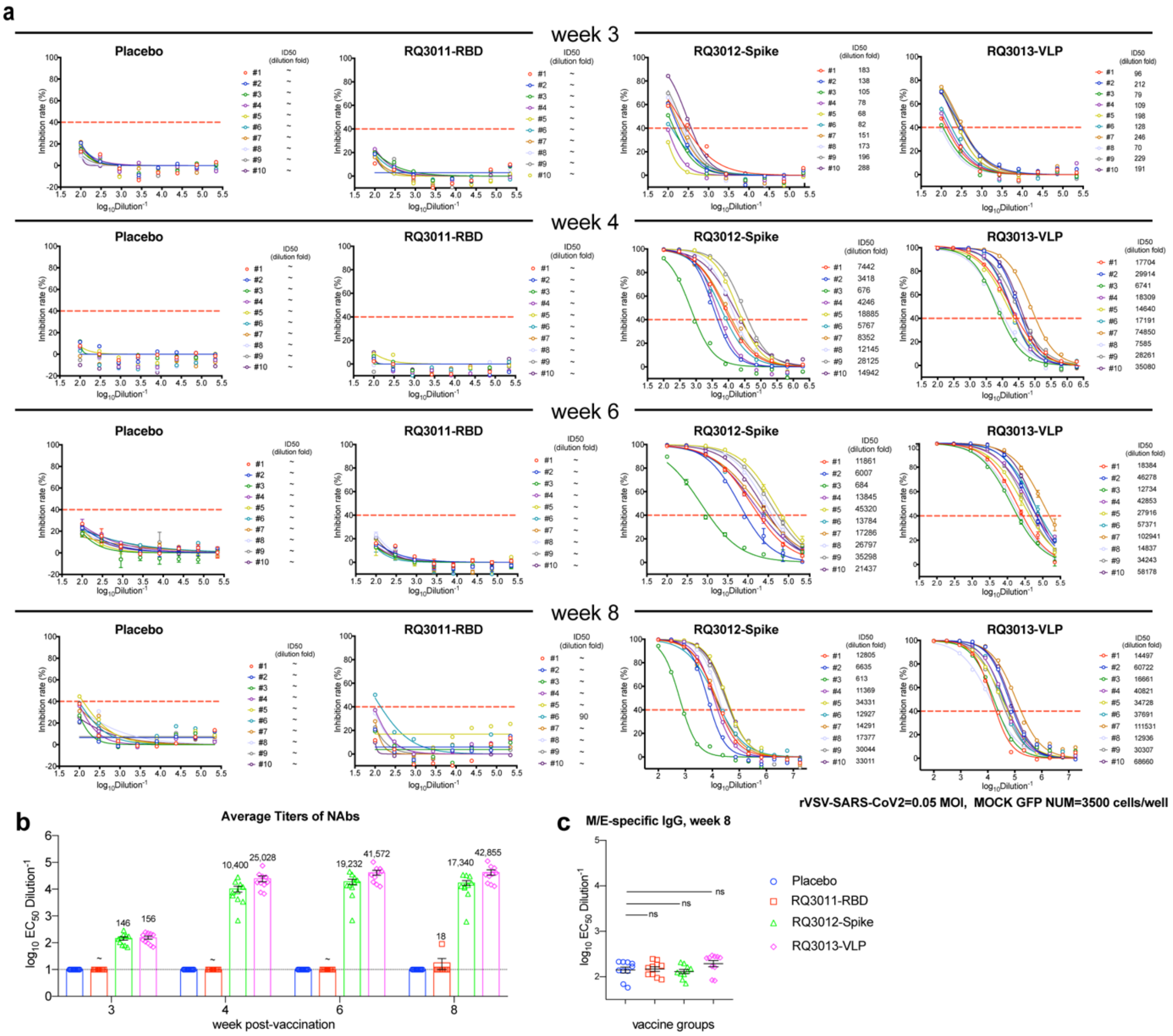

Fig. S2: Sera analysis for binding and neutralization antibodies. **a** Pseudovirus-based assay for neutralization activity of mice sera against vesicular stomatitis virus pseudotyped with SARS-CoV-2 S. Each line represents the average inhibition rate of an individual animal from one or two technical duplicates. The EC50 was analyzed by nonlinear regression, and error bars denote SEM. **b** Titers of NAbs in mice sera at weeks 3, 4, 6, and 8. **c** SARS-CoV-2 M and E protein-specific IgG in mice sera at week 8.
